# Automated determination of zinc and copper in plasma

**DOI:** 10.1155/S1463924683000516

**Published:** 1983

**Authors:** B. Sampson

**Affiliations:** Department of Chemical Pathology Charing Cross Hospital Fulham Palace Road London W6 8RF UK


Journal of Automatic Chemistry, Volume 5, Number 4 (October–December 1983), pages 207–209

### Automated determination of zinc and copper in plasma

**B. Sampson**

*Department of Chemical Pathology, Charing Cross Hospital, Fulham Palace Road, London W6 8RF*

### Introduction

The routine estimation of copper and zinc concentrations in plasma and serum is becoming a more commonly requested analysis for the management of several categories of patients. Those being maintained on total parenteral nutrition [1] are at special risk of deficiency states. There are several inborn errors of metabolism affecting copper and zinc which are readily detected by such measurements [2]. Increased urinary loss of trace metals with decreased plasma concentrations occurs in patients with liver disease, nephrotic syndrome and following injury [3 and 4].

There has been discussion about the preferred specimen for zinc and copper analysis. It is generally accepted that there is no effect on the results if serum or plasma is used for copper analysis, but large differences have been claimed for zinc analysis [5]. The suggested source of the extra zinc is from platelets damaged during the clotting process. Several recent reports have suggested no significant differences between plasma and serum zinc [6 and 7].

Another likely source of zinc contamination of plasma is haemolysis. The zinc concentration of plasma is approximately one-tenth of that of erythrocytes, whereas the copper concentrations are of the same order of magnitude. Calculation suggests that the effect of 1% haemolysis (a gross effect, readily noticed in plasma) will give an apparent increase in plasma zinc of 7·3%.

The accepted technique for copper and zinc assay in biological fluids is flame atomic absorption spectroscopy. There are several methods of sample pre-treatment in common use, i.e. trichloroacetic acid precipitation or a simple dilution with water, hydrochloric acid or butanol [8]. Spuriously high zinc values have been found using the trichloroacetic acid precipitation technique. Dilution is essential to prevent clogging of the burner due to the high solid content of serum. Other approaches recently described include 'discrete nebulization' of small volumes of samples [7], flow-injection analysis [9], and semi-automated dilution and sampling [10].

Automated continuous-flow systems, based on the Technicon Auto Analyzer have been described for the assay of calcium and magnesium concentration in plasma [11 and 12], but have not been applied to copper and zinc. Such a system is described here. The advantages over the previously mentioned techniques are that full automation and low sample consumption is possible. Continuous-flow apparatus is available in most laboratories and will be technically simpler to implement than flow-injection techniques.

### Materials and methods

### *Reagents*

Stock standards of 20 mmol/l were prepared as the metal chlorides in 0·1 mol/l hydrochloric acid, from the chloride salts dried to a constant weight at 110°C. Working standards were prepared from these (10, 20 and 30 μmol/l for zinc; 10, 20 and 50 μmol/l for copper) containing 25% (v/v) glycerol to adjust the viscosity of the standards to approximately that of plasma. The diluent used was 7·5% (w/v) butan-1-ol in deionized water, with 0·1% (v/v) of 30%-Brij 35 added.

Syringes and blood-collection tubes used were found to contain a negligible amount of zinc and copper.

### *Apparatus*

A Pye-Unicam SP1900 (Pye-Unicam, Cambridge, UK) atomic absorption spectrophotometer was used with a lean air-acetylene flame (air 5·0 l/min, acetylene 0·9 l/min) in a 10 cm single-slot burner. The analogue output was recorded on a Servoscribe chart recorder (Smiths Industries Ltd, London), using 1 mV sensitivity for zinc and 0·5 mV sensitivity for copper. An integration time of 4 s was applied to the signal to provide a degree of damping, thus giving 'smoother' response peaks and improving precision.

The continuous-flow system (see figure 1) was constructed from standard Technicon (Basingstoke, UK) Auto Analyzer components to give a sample dilution of approximately one in 10. The sample uptake into the nebulizer was modified to match the flow rate by varying the length and diameter of the connecting tubing. The wavelengths used were 213·9 nm for zinc and 324·8 nm for copper.

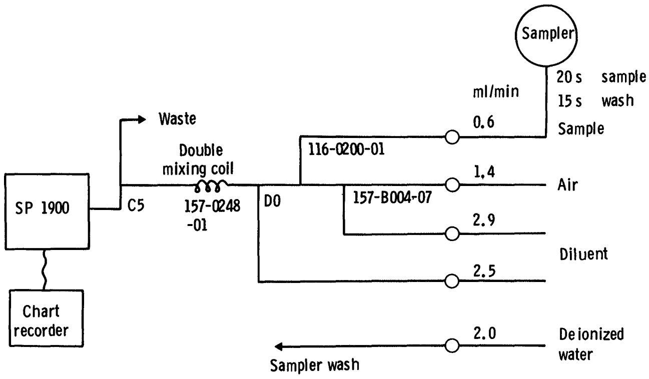

*Figure 1. Continuous-flow system for the determination of plasma zinc and copper.*

### *Procedure*

The system is operated with a sampling time of 20 s and wash time of 15 s. The chart recorder is adjusted to give a full-scale response to the highest standard used. The response is linear over the range used. A series of standards and unknown samples are loaded on to the autosampler-tray, with quality-control and drift-control samples being included. Standards are placed in triplicate and the beginning and end of each batch. Drift controls are placed after every 10 unknown samples. The internal and external quality-control samples are placed randomly among the patient samples. Internal controls are assigned target values after assays by manual volumetric dilution on at least five occasions. The acceptable range is normally ±5% of this value.

Drift can occur unpredictably in atomic absorption systems [12]. Where drift occurs, an interpolative correction can be made. Drift of more than 5% of the full-scale response over a period of 100 aspirations would lead to rejection of the batch. This is preferred to adjustment of sensitivity control during the assay due to the cumulative effect such adjustments have on the base-line and calibration slope.

### Results

### Recovery

The recovery of copper and zinc was determined by adding known amounts of standards to horse serum. A recovery of 99% for copper and 95% for zinc was found.

### Precision

Within-batch precision for the assays was estimated on five occasions using either horse serum (not the batch used for drift-control purposes), or reconstituted freeze-dried serum from a variety of sources. At least 10 replicates were analysed each time. The coefficient of variation obtained for the copper assay was 2·7±1·4% (mean±SD) in the concentration range 14–20 μmol/l and for zinc was 2·7±0·8% in the concentration range 9–12 μmol/l.

Between-batch precision was assessed in two ways: firstly by repeated analysis of pooled serum samples, and secondly by paired analysis of unknowns on two consecutive occasions. The results are shown in table 1.

### Carry-over

Carry-over was determined by the method of Broughton *et al.* [13]. The mean carry-over for copper was 1·2% and for zinc was 1·5%.

*Table 1. Between-batch precision of automated assay of zinc and copper.*

*(a) Replicates of the same sample*

| | Mean±SD[1] | Range[1] | CV | No. |
|---|---|---|---|---|
| Cu 1 | 10·96±0·52 | 10·1–12·4 | 4·7% | 14 |
| 2 | 9·48±0·56 | 8·5–10·5 | 5·9% | 17 |
| Zn 1 | 8·19±0·37 | 7·4–8·9 | 4·5% | 15 |
| 2 | 11·48±0·45 | 10·7–12·3 | 3·9% | 26 |
| 3 | 26·00±1·10 | 23·7–29·4 | 4·2% | 25 |

*(b) Paired samples*

| Concentration range[1] | Mean[1] | Difference±SD[1] | CV | No. |
|---|---|---|---|---|
| Cu <20 | 14·9 | 0·63±0·57 | 3·8% | 42 |
| <30 | 24·4 | 1·50±1·08 | 4·4% | 50 |
| >30 | 35·3 | 1·37±1·05 | 2·9% | 36 |
| Zn <10 | 7·2 | 0·50±0·39 | 5·4% | 58 |
| <20 | 13·8 | 0·69±0·59 | 4·3% | 71 |
| >20 | 22·3 | 0·80±0·68 | 2·0% | 25 |

1=μmol/l.

### Sample suitability

*Serum versus plasma*

Paired heparinized and clotted blood samples were obtained from patients attending the antenatal clinic at West London Hospital. The plasma and serum were separated at the same time within 2 h of venepuncture and stored at 4°C until analysed. The zinc concentrations found in 45 pairs of samples was (mean ±SD; μmol/l): plasma 9·8±1·7, serum 9·5±1·8.

*Contact with cells*

A more important error is introduced by the length of time the samples are left in contact with the blood cells before separating the plasma. Blood samples were taken from seven normal volunteers and each sample was divided between two heparinized tubes. One sample was immediately placed in ice and the other kept at room temperature (20°C). Aliquots of blood were centrifuged at intervals and the plasma separated for later analysis. The results are shown in table 2.

There is no effect of storage time or temperature on plasma copper concentrations. A significant increase in plasma zinc concentrations occurs in samples stored at room temperature for any length of time, but in samples stored at 4°C no significant increase in plasma zinc occurs at up to 5 h.

*Table 2. Effect of storage time and temperature on plasma, zinc and copper concentrations.*

| | Zinc | | Copper | |
|---|---|---|---|---|
| Time on cells | 4°C | 20°C | 4°C | 20°C |
| 0 | (12·4±1·2)[1,2] | | (16·1±2·8)[1,2] | |
| 1 h | −0·07±0·22[2,3] | 0·73±0·29[4] | 0·00±0·51 | −0·06±0·41 |
| 3 h | 0·09±0·35 | 1·41±0·67[4] | −0·18±0·47 | 0·00±0·42 |
| 5 h | 0·14±0·39 | 1·80±0·59[5] | −0·07±0·38 | 0·04±0·49 |
| 22 h | 1·41±0·26[5] | 4·07±1·08[5] | 0·21±0·82 | 0·18±0·72 |

1=Initial concentration of samples, μmol/l.

2=Mean SD; $N=7$.

3=Change from zero time sample, μmol/l.

4=$p<0·05$.

5=$p<0·01$.

*Haemolysis*

To test the effect of haemolysis, a small volume of haemolysate of washed erythrocyte was added to a pooled plasma sample and zinc and copper concentrations measured. No effect was found on copper concentrations. The effect of the equivalent of 0·5% haemolysis on the zinc was an increase of 1·0 μmol/l; 1·0% haemolysis caused an increase of 2·2 μmol/l. The increase in zinc found is higher than that calculated, and may be in part due to some contamination of the erythrocytes with white cells and platelets.

### Normal range

Heparinized blood was obtained from healthy subjects attending the Occupational Health Centre at Charing Cross Hospital and the plasma was separated from the cells within 1 h of venepuncture. The subjects were not suffering from any known disease, although some of the female subjects may have been taking oral contraceptives. All samples were taken between 1300 and 1500 hours. The concentrations found are shown in table 3.

*Table 3. Normal ranges for plasma, copper and zinc.*

| | Copper μmol/l | Zinc μmol/l |
|---|---|---|
| Males | 14·85±2·89[1] | 12·53±1·24 |
| $N=34$[3] | (12·1–21·9)[2] | (10·5–15·9) |
| Females | 17·98±5·08 | 11·81±1·43 |
| $N=55$ | (9·3–31·3) | (9·1–14·6) |

1 = Mean ± SD.
2 = Range.
3 = Number of subjects.

### Discussion

The method described here for the automated assay of plasma, copper and zinc is readily adaptable to most laboratories engaged in such work without the need for expensive modifications of existing apparatus. The within-batch precision achieved is below 3%, giving an acceptable precision for routine clinical assay. Addition of sodium or potassium to standards has not been found to have any beneficial effect [8] in an inter-laboratory comparison of methods. The need for viscosity matching of the samples and standards may also be relatively unimportant in some atomic absorption systems, uptake rate being dependent on nebulizer design as well as on the sample viscosity. In preliminary experiments using the SP1900 significant effects of sample viscosity on uptake rates were found.

The effect of sample storage conditions on zinc concentrations has not previously been documented. As a result of these investigations it is now recommended that plasma be the analyte of choice for zinc, and that heparinized whole blood samples be stored in ice until the plasma can be separated, but for no longer than 4–5 h. If this is not feasible (i.e. if no ice is available) the plasma should be separated within 60 min of storage at room temperature. Plasma is preferred because the time taken for clotting and clot retraction in a specimen of blood may allow the leakage described above to occur, in addition to zinc release from platelet damage during the clotting process [5]. Any sample with visible haemolysis must be considered as less than optimal for plasma zinc assay, although no such considerations need apply to copper assays.

The normal range for zinc presented here is somewhat lower than the normal ranges accepted by other workers. One factor influencing this may be the sample conditions imposed, but the known diurnal variation of plasma zinc and copper may be important [14 and 15]. Most workers have reported normal ranges on samples taken after an overnight fast. It has been shown that food intake causes the zinc content of a mid-afternoon plasma sample to be 80% of an early-morning plasma sample [16]. The values given here are on samples taken mostly after a meal and may therefore be expected to yield lower values.

### References

1. Greene, H. L., in *Zinc Metabolism: Current Aspects in Health and Disease*. Ed Brewer, G. J. and Prasad, A.S. (A.R. Liss: New York, 1977), p. 87.
2. Clayton, B. E., *Advances in Clinical Chemistry*, **21** (1980), 147.
3. Lindeman, R. D., Baxter, O. J., Yunice, A. A. and Kraikitpanich, S., *American Journal of Medical Sciences*, **275** (1978), 17.
4. Carr, G. and Wilkinson, A. W., *Clinica Chimica Acta*, **61** (1975), 199.
5. Foley, B., Johnson, S. A., Hackley, B., Smith, J. C. and Halstead, J. A., *Proceedings of the Society for Experimental Biology and Medicine*, **128** (1968), 265.
6. Kosman, D. J. and Henkin, R. I., *Lancet*, **1** (1979), 1410.
7. Makino, T. and Takahara, K., *Clinical Chemistry*, **27** (1981), 1445.
8. Taylor, A. and Bryant, T. N., *Clinica Chimica Acta*, **110** (1981), 83.
9. Rocks, B. F., Sherwood, R. A., Bayford, L. M. and Riley, C., *Annals of Clinical Biochemistry*, **19** (1982), 338.
10. Lawrence, C. B. and Phillipo, M., *Analytica Chimica Acta*, **118** (1980), 153.
11. Gochmar, N. and Givelber, H., *Clinical Chemistry*, **16** (1970), 229.
12. Nisbett, J. A. and Owen, J. A., *Clinica Chimica Acta*, **92** (1979), 367.
13. Broughton, P. M. G., Gowenlock, A. W., McCormack, J. J. and Neill, D. W., *Annals of Clinical Biochemistry*, **11** (1974), 207.
14. Lifschitz, M. D. and Henkin, R. I., *Journal of Applied Physiology*, **31** (1971), 88.
15. Marotta, S. F., Lanuza, D. M. and Hills, L. G., *Hormone and Metabolic Research*, **6** (1974), 329.
16. Richards, B., Flint, D. M., and Wahlquist, M. L., *Nutrition Reports International*, **23** (1981), 939.

### FORTHCOMING PAPERS

1984's issues will include:

*S. Wilson and A. Green* on 'An assessment of the IQAS discrete analyser for routine water chemistry laboratory use'

*H.-J. Stan and H. Goebel* on a BASIC program to combine data from two selective detectors and its application for screening of pesticides in residue analysis

*C. Ferré et al.*: 'An evaluation of the Hitachi 705 analyser'

*R. Stanley*: 'Automation in analytical chemistry—from rule of thumb to fully automated methods. Some philosophies and social consequences'

*S. Oldfield et al.*: 'An evaluation of the Mitsubishi GL-101 glucose analyser'

*P. J. Gemperline et al.*: 'DISNET: a distributed instrument network'

